# Integrating Functional Cognitive Rehabilitation With Activity-Based Stroke Telerehabilitation: Intervention Design and Development Study

**DOI:** 10.2196/84627

**Published:** 2026-10-27

**Authors:** Patricia Finetto, Michelle L Woodbury, Julianne Laura, Kelly Rishe, Stephanie Garner, Lisa M McTeague, Deena Schwen Blackett, Elizabeth W Twamley, Stephanie Aghamoosa

**Affiliations:** 1 Department of Health Sciences & Research, College of Health Professions, Medical University of South Carolina, 77 President Street, Charleston, SC, 29425, United States, 1 8437922956; 2 Department of Psychiatry and Behavioral Sciences, College of Medicine, Medical University of South Carolina, Charleston, SC, United States; 3 Ralph H. Johnson Veterans Affairs Medical Center, Charleston, SC, United States; 4 School of Communication Sciences and Disorders, College of Health Professions, University of Central Florida, Orlando, FL, United States; 5 VA San Diego Healthcare System, San Diego, CA, United States; 6 Department of Psychiatry, University of California San Diego, San Diego, CA, United States

**Keywords:** telerehabilitation, cognition, cognitive rehabilitation, occupational therapy, neuropsychology, stroke

## Abstract

**Background:**

Many stroke survivors experience chronic cognitive deficits that, together with motor impairments, negatively impact well-being and daily function. Critically, poststroke cognitive deficits can interfere with successful engagement in rehabilitation programs aimed at improving participation in home and community activities. To address this issue, we developed a new telehealth intervention that integrates brief, functional cognitive rehabilitation (COG) into an existing upper extremity-focused, activity-based occupational therapy (OT) telerehabilitation program.

**Objective:**

To describe the development and specification of a telehealth intervention (COG+OT) that integrates functional cognitive rehabilitation into activity-based stroke telerehabilitation.

**Methods:**

COG+OT was developed using a multi-disciplinary, theory-informed process guided by principles of functional cognitive rehabilitation. It is reported according to the Template for Intervention Description and Replication checklist for rehabilitation (TIDieR-Rehab). The intervention was developed by a team with expertise in stroke rehabilitation from the fields of OT, neuropsychology, clinical psychology, and speech-language pathology. Cognitive rehabilitation content was adapted from an evidence-based compensatory strategy program and condensed into a brief “primer” format. The COG component was iteratively integrated into an existing upper-extremity, activity-based OT telerehabilitation program to support engagement and transfer of strategies to real-world functional tasks. In this paper, we describe the intervention structure, content, delivery, and tailoring procedures.

**Results:**

COG+OT is an 8-week, 13-session telehealth intervention delivered in three phases: (1) cognitive strategy training (“primer”; sessions 1-4), (2) review and guided application of strategies within OT activities (sessions 5‐10), and (3) integration of strategies to support generalization to everyday contexts (sessions 11-13). The intervention combines individualized, strategy-based cognitive rehabilitation with goal-oriented OT using a titrated approach. Patient-facing materials, structured session workflows, and therapist-guided metacognitive strategies support tailoring and real-world application.

**Conclusions:**

COG+OT is a theory-informed, user-centered intervention that provides a structured approach for integrating functional cognitive rehabilitation into stroke telerehabilitation. This work establishes a well-specified, replicable intervention model and supports future feasibility, effectiveness, and implementation studies.

## Introduction

Poststroke cognitive deficits occur in up to 70% of stroke survivors, most commonly in the domains of memory, attention, and executive function [1]. These multifaceted cognitive deficits are consequential for stroke recovery, as they are associated with lower mood and quality of life, reduced participation in daily activities, and more protracted disability [2,3]. Cognitive rehabilitation interventions are effective at remediating cognitive dysfunction across many clinical conditions [4], and growing evidence indicates that functional cognitive rehabilitation, defined as strategy-based training aimed at enhancing real-world cognitive functioning, is particularly useful for improving everyday functioning poststroke [5]. However, cognitive rehabilitation is underutilized in stroke care due to prioritization of physical over psychosocial and cognitive recovery [6], limited access to cognitive rehabilitation services, lack of a well-defined replicable intervention model, and the burden of conventional multiweek treatment schedules. Furthermore, it is typically delivered as a stand-alone intervention, separate from other therapies in which cognitive strategies could be practiced and applied, creating a missed opportunity for integrated rehabilitation that enhances generalization of skills into daily life routines. With the increasing recognition of cognitive health as critical for functioning and quality of life, poststroke therapies combining physical and cognitive rehabilitation are an urgent need.

In prior work, we developed a telerehabilitation occupational therapy (OT) program that integrates upper-extremity task practice [7] with metacognitive strategy training (ie, teaching individuals to plan, monitor, and evaluate their own performance) based on the Cognitive Orientation to daily Occupational Performance approach [8]. In a recent study in adult stroke survivors, we showed that this activity-based telerehabilitation OT program improved paretic upper extremity motor skills, activity performance, self-efficacy, and skill generalization [9]. However, because this program is cognitively demanding, with metacognition components (ie, the process of thinking about one’s own thinking and adjusting accordingly [10]) that require collaborative goal setting, self-reflection, error analysis, and problem solving, stroke survivors with cognitive deficits may struggle to fully engage and benefit. This challenge served as the motivation for integrating functional cognitive rehabilitation into this activity-based OT program.

This new intervention, called COG+OT, pairs a brief, functional cognitive rehabilitation “primer” (COG; this abbreviation specifically refers to the brief cognitive rehabilitation component of this intervention) with the previously developed activity-based OT program. The rationale for combining these approaches is based on evidence that compensatory cognitive strategies enhance the attention, metacognition, and goal management skills needed for therapy engagement [5], and cognitive strategies generalize most effectively when practiced within meaningful everyday activities [4]. The cognitive rehabiliation intervention we adapted was based on compensatory cognitive training approaches that have demonstrated benefits for cognition, functional capacity, and quality of life in randomized controlled trials including individuals with acquired brain injury and other neuropsychiatric conditions [11]. Our central hypothesis is that delivering COG before and alongside OT may enhance cognitive readiness, thereby supporting engagement in activity-based OT and yielding greater gains in real-world activity performance and participation.

We recently completed a single-arm pilot feasibility trial of 8-week COG+OT with 20 adults with chronic stroke and upper extremity paresis. Initial pilot testing supported the feasibility and acceptability of the intervention, as reported previously [12]. Given these findings, the purpose of the current paper is to describe the COG+OT intervention to support transparency, replication, and future intervention evaluation. The primary objective of this manuscript is to detail the conceptualization, design, and specification of the COG+OT intervention using systematic reporting via the Template for Intervention Description and Replication checklist for rehabilitation trials (TIDieR-Rehab) [13].

## Methods

### Ethical Considerations

The intervention was developed and tested in a pilot feasibility trial that was approved by the Medical University of South Carolina Institutional Review Board (Pro00136303) and registered with ClinicalTrials.gov (NCT06555302). All participants provided informed consent.

### Intervention Development Process

#### Theoretical Rationale

The intervention embeds functional cognitive rehabiliation into an existing 9-session stroke telerehabilitation program [9] (OT) by using brief COG sessions as a “primer” to boost engagement in and benefit from OT (Figure 1A). Compensatory cognitive training was selected based on theoretical models emphasizing that functional cognitive improvement is best achieved through metacognitive strategy training, habit learning, environmental scaffolding (ie, external supports that facilitate performance), and contextualized practice rather than isolated restorative exercises [4,14]. These models highlight mechanisms such as teaching compensatory strategies (ie, working around deficits and capitalizing on intact abilities), enhancing self-regulatory and metacognitive processes, and applying strategies in meaningful real-world contexts to promote transfer. These principles align closely with a person-centered, goal-oriented, activity-based OT program.

**Figure 1.**
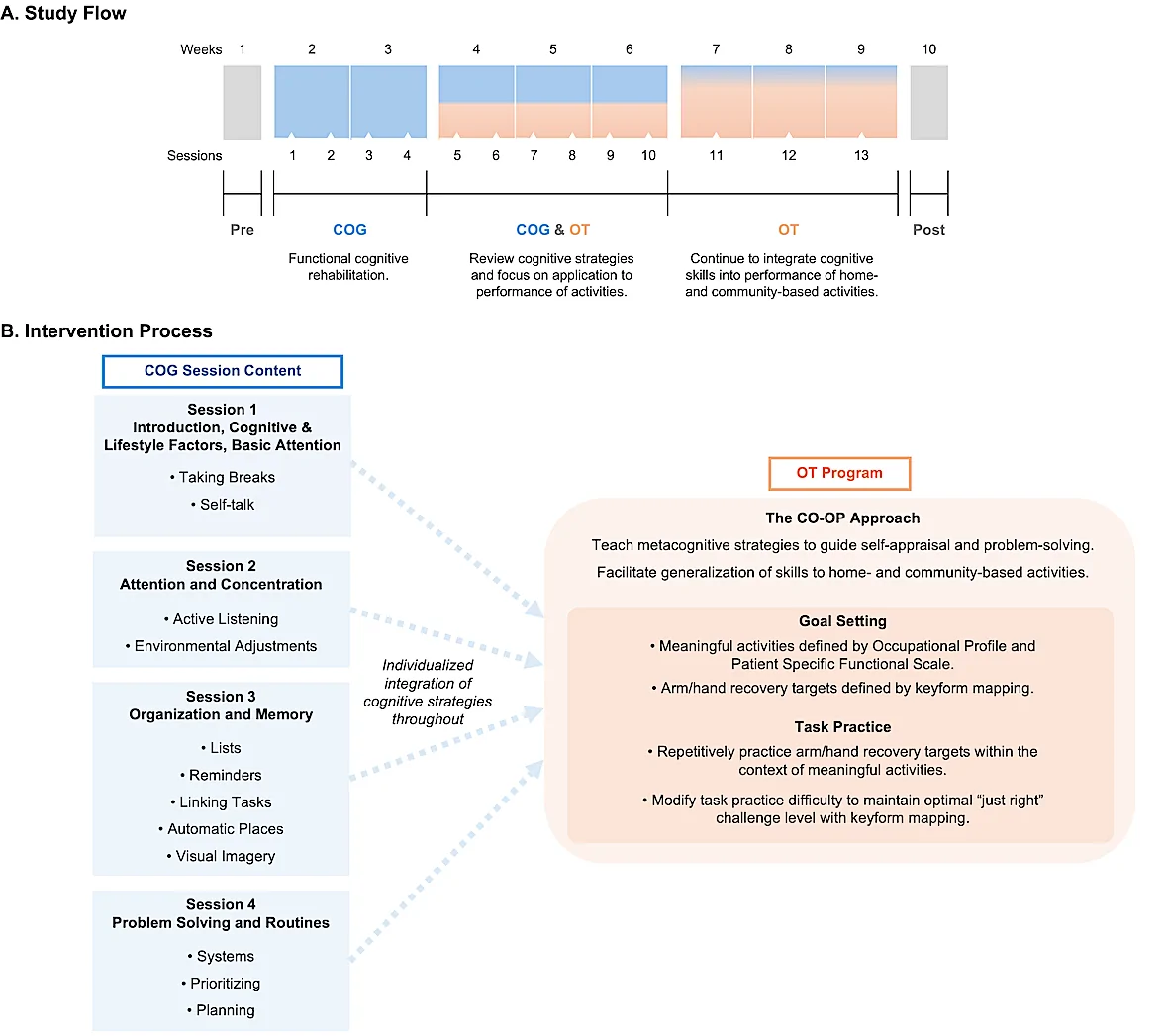
Study flow and intervention overview. Panel A shows the procedures conducted during each week of the study, including outcomes assessments (“Pre” and “Post”), and the 3-phase intervention delivered across 13 sessions. Panel B lists the core topics covered in each of the 4 COG sessions (with additional optional topics such as calendar use available, when relevant), depicts the integration of these cognitive strategies into the pre-existing OT program, and provides an overview of the key components of the OT program. COG: cognitive rehabilitation; CO-OP: Cognitive Orientation to Daily Occupational Performance; OT: occupational therapy.

#### Relevance to the Existing OT Program

The existing OT program [9] is cognitively demanding, combining (1) upper-extremity task practice within the context of individualized activity goals identified using the American Occupational Therapy Association (AOTA) Occupational Profile [15] and the Patient-Specific Functional Scale [16] and (2) metacognitive strategy training based on the Cognitive Orientation to Daily Occupational Performance approach [8] (Figure 1B). Therapists encourage participants to use the “Goal-Plan-Do-Check” global cognitive strategy as a framework to plan, perform, and evaluate task performance. These elements require cognitive flexibility, self-monitoring, problem-solving, and sustained attention. Thus, the COG “primer” was intentionally designed to bolster functional cognition so participants could more effectively engage in the OT program. Specifically, COG session content focuses on learning and applying attention, organization, memory, and problem-solving strategies to facilitate performance of real-world tasks. Examples include planning a modified approach to challenging daily tasks, eliminating distractions to improve attention during functional activities, using reminders to implement learned strategies while completing activities, and engaging in a structured reflection process to self-analyze performance during the OT sessions.

#### Conceptual Intervention Design

Unlike conventional cognitive rehabilitation programs delivered over multiple weeks, we condensed the content into a brief format that was delivered using a titrated model to reduce participant burden and facilitate integration of cognitive strategies directly into OT. This design aimed to optimize generalization by first introducing strategies, then applying them during functional activities. Titration occurred across three phases (Figure 1A): (1) individualized cognitive strategy instruction (sessions 1-4), (2) brief review and application of strategies within activity-based OT (sessions 5-10), and (3) continued integration of strategies into OT to promote generalization and habit formation (sessions 11-13). Personalized homework assigned at each session supported skill generalization to home and community environments.

#### Adaptation of COG Materials

COG materials were adapted from the 8-week Motivationally Enhanced Compensatory Cognitive Training for Mild Cognitive Impairmentprogram (M Huckans, et al; Motivationally Enhanced Compensatory Cognitive Training for Mild Cognitive Impairment [ME-CCT for MCI]; unpublished treatment manual; 2013), an iteration of evidence-based Compensatory Cognitive Training and Cognitive Symptom Management and Rehabilitation Therapy (EW Twamley, et al; Cognitive Symptom Management and Rehabilitation Therapy [CogSMART] for Traumatic Brain Injury; unpublished treatment manual; 2010). Materials for ME-CCT/CogSMART are available on the CogSMART website [17] and were adapted with permission for research use and acknowledgment of the original sources. These interventions emphasize compensatory cognitive strategies and habit learning and have been delivered via telehealth in prior research studies and clinical care settings [18-20]. Study team members (occupational therapists [PF, SG, JL, KR, MLW] and a neuropsychologist [SA]) reviewed the ME-CCT manual, selected stroke-relevant content, and condensed it into four 1-hour sessions (Figure 1B). Intervention delivery was designed to be individualized in terms of time spent on each cognitive strategy (based on relevance), focus on improving vs optimizing skills (based on impairment level), and application to activities/settings (based on context and goals).

The intervention was developed by a multidisciplinary rehabilitation team with expertise in cognitive rehabilitation and stroke telerehabilitation, including a clinical neuropsychologist (SA), occupational therapists (PF, SG, JL, KR, MLW), a clinical psychologist (LMM), and a speech-language pathologist (DSB). The neuropsychologist led development of the COG component, including strategy selection and adaptation (SA in consultation with EWT), while the OT team led integration of cognitive strategies into the existing activity-based telerehabilitation program. The speech-language pathologist guided adaptation of patient-facing materials to accommodate individuals with mild-to-moderate aphasia.

## Results

The intervention development process resulted in COG+OT, an 8-week, 13-session telerehabilitation program delivered across three phases: (1) cognitive strategy training (sessions 1-4), (2) guided application of cognitive strategies within activity-based OT (sessions 5-10), and (3) continued integration and generalization of strategies during OT sessions (sessions 11-13; Figure 1). The completed TIDieR-Rehab checklist [13] for the COG+OT intervention is reported in Checklist 1, and details are provided in the following sections.

### Personnel

COG+OT is designed to be delivered by licensed occupational therapists with training in stroke rehabilitation and telehealth. In this implementation, therapists were experienced in both domains and received structured training in the COG component and its integration with the OT program as well as ongoing supervision via weekly group case consultation with lead clinicians (SA, MLW).

### Setting

COG+OT is designed for delivery via synchronous telehealth using a Health Insurance Portability and Accountability Act (HIPAA)–compliant videoconferencing platform (in this study, doxy.me), enabling participation from home and community environments. This intervention uses participants’ real-world contexts by incorporating personally meaningful activities and readily available household spaces and materials into therapy sessions. Therapists ensure camera positioning to allow clear visibility of movements and task performance. Assistance and training with the videoconferencing technology is provided as needed. When appropriate, caregivers may assist with technology setup or safety monitoring. This home-based delivery model is intended to enhance ecological validity, accessibility, and generalization of cognitive and motor strategies to everyday activities.

### Target Population

The COG+OT intervention is intended for adults with subacute and chronic stroke experiencing upper-extremity motor impairment and co-occurring cognitive challenges who are able to participate in telehealth-based rehabilitation. The COG+OT model is intended to be broadly applicable to stroke survivors who would benefit from integrated cognitive and activity-based rehabilitation and is therefore applicable to individuals with mild-to-moderate cognitive deficits in various domains (eg, attention, memory, executive function). The intervention is also designed to accommodate individuals with mild-to-moderate aphasia through the use of adapted, “aphasia-friendly” materials [21,22] and multimodal communication supports (developed in consultation with the speech-language pathologist [DSB]). Consideration for safety (eg, fall risk) and the potential need for caregiver assistance are incorporated into intervention delivery.

### Intervention Materials

#### Measures Used to Guide Individualized Delivery of the Intervention

##### Occupational Profile

At the pretreatment assessment visit, therapists complete an AOTA Occupational Profile [15] via semistructured interview to identify participants’ occupational history, patterns of daily living, values, and participation priorities. Insights obtained through the occupational profile are used to inform individualized goal setting and intervention planning. As participants complete the 4 initial COG sessions, the occupational profile is used to guide collaborative conversations to help the therapist gain a deeper understanding of participants’ routines, habits, and roles in relation to their cognitive functioning.

##### Patient-Specific Functional Scale

At the pretreatment assessment visit, therapists administer the Patient Specific Functional Scale [16] to identify each participant’s activity goals. Participants are asked to generate up to 5 meaningful activities that have been made difficult due to upper extremity motor impairment since having a stroke. Participants then rate their ability to perform each activity on a 0‐10 scale, with higher numbers indicating better self-perceived performance. Throughout the OT program, sessions focus on optimizing participant performance in the context of these goals. Therapists regularly reference and revisit the goals with the participant when identifying target activities for the treatment sessions. This scale is readministered at the posttreatment assessment to assess change in self-perceived performance of individualized activity goals.

##### Upper Extremity Motor Assessment

Prior to beginning the phase-2 COG+OT sessions (at the end of session 4), the participant’s hemiparetic arm/hand impairment is measured using the Fugl-Meyer Assessment of the Upper Extremity [23], which is the gold-standard assessment of poststroke arm/hand motor impairment. In prior work, we modified this measure for use in telerehabilitation [9], reducing it to 22 items that can be assessed virtually. Administration involves the therapist observing the participant’s performance of 22 movements (ie, moving the hemiparetic arm/hand through a series of postures), rating each on a 3-point scale (0=unable to perform, 1=partially performed, 2=near normal performance). Items are summed to a total score between 0-44 points, with lower scores indicating more severe motor impairment. Using a procedure described in detail previously [7], the participant’s Fugl-Meyer item performance ratings are mapped onto an item hierarchy (ranging from easy to difficult) to indicate person-specific arm/hand movements that are optimally challenging targets for task practice therapy. This process, termed “keyform recovery mapping” (or “recovery ruler”) [24], is an emerging evidence-based method that operationalizes how item difficulty and performance are used to select individualized treatment targets. To monitor progress and refine individualized movement targets, the Fugl-Meyer Assessment is readministered and mapped to the keyform at the intervention midpoint (end of session 7) and at the end of phase-2 COG+OT sessions (end of session 10). To evaluate change in motor function, it is administered again at the posttreatment assessment.

### Materials Used to Implement the Intervention

#### Session Tracking Notes

Therapists document session activities and participant progress using study-developed Session Tracking Notes in REDCap. The notes differ slightly in structure across the 3 intervention phases, but each session’s note contains the following content: (1) Subjective (participant report and direct quotes), (2) Objective Session Content (checklist of completed content, documentation of functional task practice activities, and notes on activity grading per therapist report), (3) Assessment (checklists capturing therapists’ ratings of participant response to the intervention and barriers to progress), and (4) Notes/Follow-Up Plan, including assigned home activity program (therapist narrative). The narrative portions of the Subjective, Assessment, and Plan sections reflect best-practice rehabilitation documentation procedures.

#### COG Session Manual

A manual was developed to guide therapists during COG sessions (sessions 1-4). The manual outlines session procedures and content to be covered, provides examples that could be shared with participants, and includes guidance for adjusting the complexity of information to support participant comprehension in the presence of cognitive and/or language impairment.

#### Patient-Facing COG Worksheets

For each COG session, we created 1‐2-page patient-facing worksheets, written at an eighth-grade reading level, that can be screen-shared, mailed, and/or emailed to the participant to guide discussion. Each session’s worksheet contains (1) an introduction section that included descriptions of core concepts, definitions of key terms, and educational content followed by (2) a “Let’s Practice” section containing real-life examples, space for self-reflection, and discussion prompts focused on functional application of cognitive strategies. Participants (and/or caregivers when present) are encouraged to take notes and regularly reference the worksheets outside of sessions.

### Intervention Process

#### Overview

The intervention consists of 13 one-on-one telehealth sessions (45‐60 minutes each) over 8 weeks (Figure 1A). The schedule is 2 sessions per week for the first 5 weeks, followed by 1 session per week for the final 3 weeks. The reduction in session frequency during the latter phase is intended to promote greater independence and encourage skill generalization and transfer to real-world activities. To accommodate participant scheduling challenges, we allow make-up sessions to be added to any intervention week and for sessions to extend into one additional week (9 weeks total), if needed. The approximate proportion of each session allocated to COG vs OT activities varies over the 3 phases of the intervention (described in the following sections).

#### Sessions 1-4 (COG-Only)

Each COG session focuses on specific topics, listed in Figure 1B. Although the COG content follows a structured sequence, it is intentionally flexible to allow therapists to individualize the emphasis based on participant needs (eg, relevance, interest). Similarly, the use of cognitive strategies to compensate for deficits vs optimize intact abilities is personalized based on cognitive impairment level and contextual demands. Using the patient-facing worksheets via screensharing, therapists first introduce the session topic and review the associated compensatory strategies. The therapist and participant then collaboratively identify which strategies are most relevant to the participant’s goals and cognitive challenges. Remaining session time focuses on practicing these strategies and discussing how they might be applied in daily activities. Personalized homework is assigned to reinforce real-world use of cognitive strategies and is reviewed with the therapist at the beginning of each following session. Throughout these sessions, therapists make note of strategies that emerge as most relevant or useful for each participant. Additional worksheets covering topics beyond the core sessions (eg, calendar use, additional memory strategies) are available and may be incorporated when relevant and if time permits. No new strategies are introduced after session 4.

#### Sessions 5–10 (COG+OT)

Each session begins with a brief review of the participant’s use of learned cognitive strategies in their daily lives (10-15 minutes). The remainder of the session focuses on direct application of the strategies during OT activities. During the first COG+OT session (session 5), the telerehabilitation version of the Fugl-Meyer Assessment of the Upper Extremity keyform (described in *Intervention Materials*) is completed, and the therapist and participant review item performance scores to identify optimally challenging hemiparetic upper extremity movements to target in therapy. Together with the participant’s goals from the Patient Specific Functional Scale and Occupational Profile, this information guides selection of meaningful in-home activities. Participants then practice these activities while being coached by the therapist using metacognitive strategy training and guided discovery based on the Cognitive Orientation to Daily Occupational Performance approach [8] (Figure 1B). Therapists use the “Goal-Plan-Do-Check” global cognitive strategy as a framework (ie, participants identify the task “goal,” verbalize the “plan” to achieve the goal, attempt to “do” the task, and then self-reflect to “check” their own overall performance) while encouraging the participant to apply relevant cognitive strategies to support task completion. See Figure 2 for an example session.

**Figure 2.**
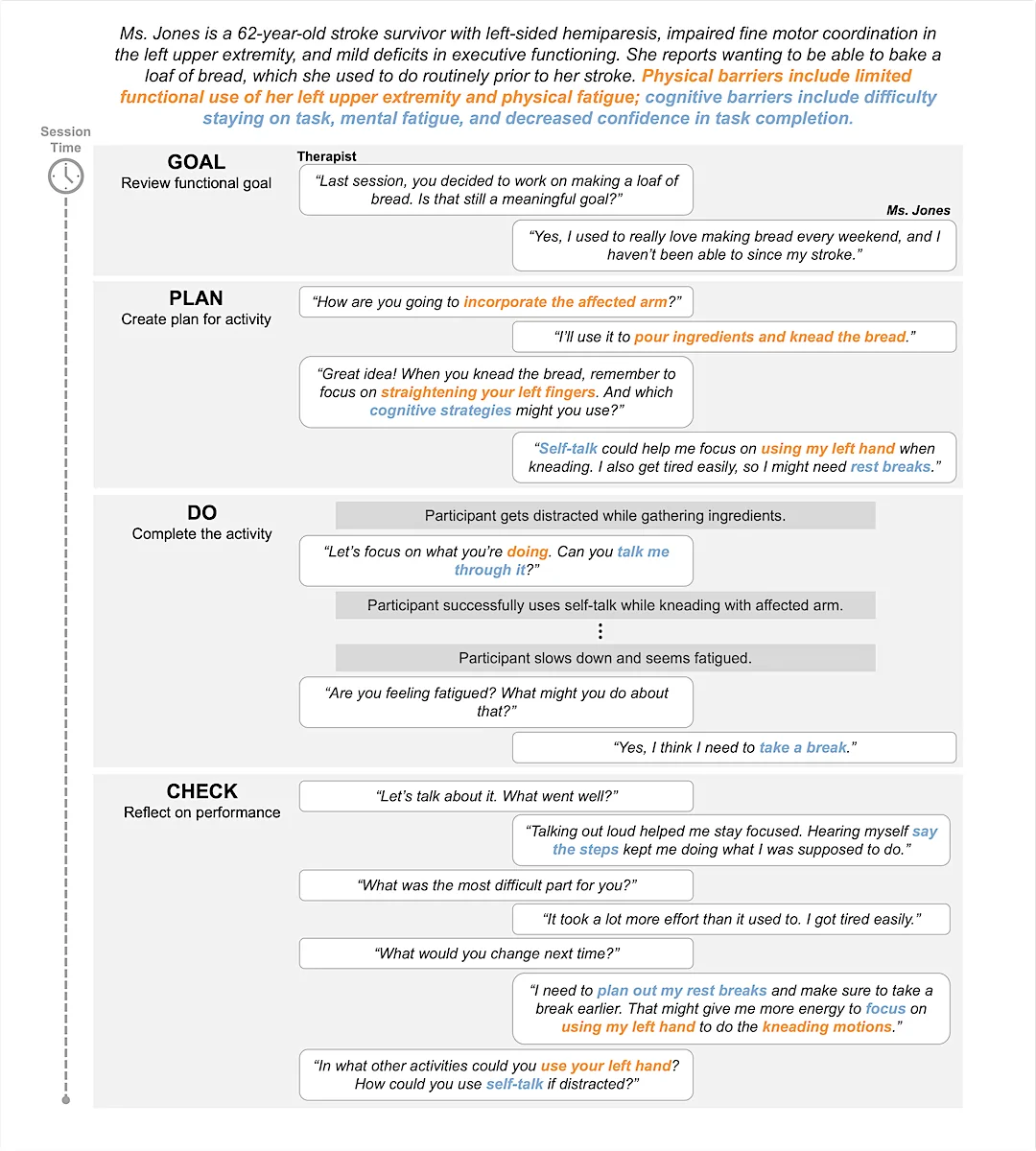
Example COG+OT session (sessions 5-13). A representative scenario illustrates how individualized goals, motor targets, and cognitive strategies are integrated during task practice, with therapist-guided coaching supporting performance and reflection.

#### Sessions 11‐13 (OT With Integrated COG)

Cognitive strategies are no longer explicitly reviewed during these sessions, but participants are encouraged to continue applying the strategies during OT functional task practice (using the same procedures as sessions 5‐10). For example, a participant who had identified self-talk as a helpful strategy in earlier sessions would be coached to verbalize task steps during OT functional task practice to enhance attention and performance (see Figure 2). Therapists reinforce effective cognitive strategy use during target activities and facilitate generalization of new cognitive and motor skills to home and community contexts.

### Intervention Fidelity

Multiple procedures are implemented to support consistent delivery of the intervention. All therapists receive training in both the COG component from the study neuropsychologist (SA) and in the OT program from the lead occupational therapist (MLW). A treatment manual was developed to standardize procedures, and standardized session workflows are used to guide intervention delivery and promote consistency across therapists. Intervention session activities and participant progress are documented using Session Tracking Notes (described in *Intervention Materials*) within a secure data management platform (ie, REDCap). These data are used to track progress and guide subsequent sessions. Protocol deviations, barriers to progress, and adverse events are systematically recorded in REDCap and reviewed. If the planned content is not fully covered in the allotted time or when additional practice is needed, this is noted in REDCap and addressed in subsequent sessions to ensure each participant is exposed to all the core material.

## Discussion

We developed COG+OT, a theory-informed intervention that integrates brief, functional cognitive rehabilitation into activity-based stroke telerehabilitation. This work addresses the well-documented challenge in poststroke care of siloed services for cognitive vs motor rehabilitation and lack of a well-defined multidisciplinary intervention protocol, which limits opportunities to apply cognitive strategies to meaningful activities and contexts. By embedding cognitive strategy training within meaningful, goal-directed activities, COG+OT is intended to support both engagement in rehabilitation and transfer of skills to everyday life.

An innovative design feature is the titrated integration of cognitive and motor components across 3 phases. The initial “primer” phase introduces compensatory cognitive strategies in a structured but individualized format, allowing participants to develop awareness and understanding of their cognitive challenges and strategies for addressing them. This is followed by guided application of these strategies within activity-based OT sessions and then continued integration to promote skill generalization into daily activities to form routines and habits. This progression aligns with contemporary models of functional cognitive rehabilitation, which emphasize metacognitive strategy training, contextualized practice, and repeated application across settings to facilitate generalization to real-world activities [4,14].

The use of a brief cognitive “primer” is intended to reduce burden while retaining core elements of evidence-based compensatory approaches, including personalization and strategy use in everyday contexts. Crucially, the COG sessions make cognitive strategies explicit for participants, complementing the implicit use of these strategies within the therapists’ metacognition-based coaching. For example, therapists may introduce visual imagery during COG sessions as a strategy for planning and organization, then prompt participants to visualize each step of a task during OT sessions to support more accurate and efficient motor performance. Although the COG component draws from evidence-based materials (ie, ME-CCT/CogSMART), there are several other established cognitive rehabilitation programs that could have been considered. Approaches such as the Multicontext Approach, Goal Management Training, and other functional cognitive interventions [5,25,26] also include theoretically grounded elements (eg, metacognitive strategy training, guided practice, contextualized transfer) that may further strengthen components of COG+OT. Future iterations of the intervention may therefore benefit from selectively integrating features from these programs, particularly those that support generalization. In addition, explicit incorporation of motivational interviewing techniques may further enhance engagement, goal alignment, and sustained strategy use in daily life.

COG+OT was designed for real-world implementation through its telehealth delivery and use of participants’ home environments to enhance ecological validity. Structured yet flexible materials support intervention fidelity while allowing for individualized tailoring. Additional features, including patient-facing worksheets, aphasia-friendly materials, and optional caregiver involvement increase inclusivity and make COG+OT amenable to implementation in a variety of settings (eg, outpatient clinics, community-based rehabilitation, hybrid telehealth models). Moreover, because compensatory cognitive strategies and metacognitive training are relevant across many neurologic and medical conditions, there is strong potential to adapt COG+OT for use in other populations (eg, individuals with traumatic brain injury, mild cognitive impairment, or other chronic conditions in which functional cognition supports daily participation) or clinical services (eg, speech therapy, physical therapy). Therefore, a key next step is to evaluate the feasibility, effectiveness, and implementation of COG+OT across diverse populations and settings. To support future dissemination, additional work is needed to develop standardized training and fidelity-monitoring procedures alongside evaluation of scalable delivery models and factors influencing real-world adoption.

Initial pilot testing supported the feasibility and acceptability of COG+OT telerehabilitation for stroke survivors, as reported previously [12]. The current manuscript extends that work by providing a detailed description of the intervention’s theoretical basis, structure, materials, and delivery procedures to support replication and ongoing research. Analyses are underway to examine preliminary effects on cognitive, psychosocial, and participation-related outcomes, while qualitative feedback from participants and therapists will inform continued refinement of the intervention. Although aphasia-friendly adaptations were incorporated into COG+OT, additional research including greater representation of people with aphasia will be needed to evaluate their appropriateness. Future studies will also examine the added value of integrating functional cognitive rehabilitation into activity-based telerehabilitation, informing the design of future randomized controlled efficacy trials.

This paper focused on the systematic specification of the COG+OT intervention, including its theoretical basis, structure, and delivery procedures. Overall, COG+OT provides a structured and adaptable model for integrating functional cognitive rehabilitation into stroke telerehabilitation. This development work lays the groundwork for future refinement, evaluation, and broader implementation of COG+OT as an evidence-based, manualized intervention.

## Supplementary material

10.2196/84627 Checklist 1 TIDieR-Rehab checklist.

## Abbreviations

AOTA: American Occupational Therapy Association
COG: cognitive rehabilitation
CogSMART: Cognitive Symptom Management and Rehabilitation Therapy
HIPAA: Health Insurance Portability and Accountability Act
ME-CCT: Motivationally Enhanced Compensatory Cognitive Training
OT: occupational therapy
TIDieR-Rehab: Template for Intervention Description and Replication checklist for rehabilitation trials
